# Higher cardiorespiratory fitness is strongly associated with lower cardiovascular risk factors in firefighters: a cross-sectional study in a German fire brigade

**DOI:** 10.1038/s41598-021-81921-1

**Published:** 2021-01-28

**Authors:** Markus Strauss, Peter Foshag, Ulrich Jehn, Anna Brzęk, Henning Littwitz, Roman Leischik

**Affiliations:** 1 Department of Cardiology, Sector Preventive Medicine, Health Promotion, Faculty of Health, School of Medicine, University Witten/Herdecke, 58095 Hagen, Germany; 2 Department of Cardiology I- Coronary and Peripheral Vascular Disease, Heart Failure Medicine, University Hospital Muenster, Cardiol, 48149 Muenster, Germany; 3 Division of General Internal Medicine, Nephrology and Rheumatology, Department of Medicine D, University Hospital of Muenster, 48149 Muenster, Germany; 4 Department of Physiotherapy, Chair of Physiotherapy, School of Health Sciences, Medical University of Silesia, Katowice, Poland

**Keywords:** Risk factors, Health occupations, Occupational health, Public health

## Abstract

Previous studies have shown significant cardiovascular risks in firefighters and that they suffer from cardiovascular events, especially on duty. Otherwise, adequate cardiorespiratory fitness is considered to have a protective effect in reducing cardiovascular complications. Therefore, the study aimed to evaluate the association between cardiorespiratory fitness and cardiovascular risks factors in firefighters. We enrolled ninety-seven male German firefighters in this cross-sectional study of cardiorespiratory fitness and cardiovascular risk factors. We used spiroergometry testing to estimate oxygen consumption to determine cardiorespiratory fitness and to calculate metabolic equivalents. We evaluated cardiovascular risk factors included nicotine consumption, lipid profiles, body composition, resting blood pressure, and heart rate. We evaluated cardiovascular risk factors included nicotine consumption, lipid profiles, body composition, resting blood pressure and heart rate. The comparison of association between cardiorespiratory fitness and cardiovascular risk factors was performed by using χ^2^-test, analysis of variance, general linear regression with/without adjustment for age and body mass index (BMI). This study demonstrated a strong association between lower cardiovascular risk factors and higher cardiorespiratory fitness. There were significantly lower values for BMI, waist circumference, body fat percentage and resting systolic blood pressure, triglycerides, and total cholesterol (all p < 0.0443, age-adjusted) with increased cardiorespiratory fitness. Only 19.6% (n = 19) of the examined firefighters were classified as “fit and not obese”, 48.4% (n = 47) were “low fit and not obese” and 30.9% (n = 30) were “low fit and obese”. The results clarify that increasing cardiorespiratory fitness is a fundamental point for the reduction and prevention of cardiovascular complications in firefighters. It could be demonstrated, especially for central risk factors, particularly BMI, waist circumference, sytolic resting blood pressure and triglyceride values. Therefore, firefighters should be motivated to increase their cardiorespiratory fitness for the beneficial effect of decreasing cardiovascular risk profile.

## Introduction

Firefighting is known to be a physically demanding and dangerous occupation. Firefighters are exposed to high physical and psychological stress in their daily work^1,2^.

They often have to manage physically strenuous situations in which a maximal cardiorespiratory performance is essential^3^. Good cardiorespiratory performance is crucial for firefighters’ safety and protects them from the development of cardiovascular risks and diseases^4^.

Extensive epidemiological studies have shown that physical activity is one of the most important prevention factor for protection against cardiovascular diseases worldwide^5–8^. Physical fitness protects against obesity and diabetes mellitus^9–11^. On the other hand, physical inactivity is responsible for the development of breast and colon cancer in up to 25% and the development of diabetes mellitus in up to 27% of cases^12^. Physical inactivity causes up to 30% of coronary artery disease cases^12^. Research in the United States has shown that firefighters have identical cardiovascular risk profiles as compared to the overall population^13^. Despite the similar risk profile, the number one cause of death for on-duty firefighters in the United States is cardiovascular disease^14^. One of the essential pathogenetic risk factors in this occupational group is arterial hypertension^15^.

Peate et al.^16^ describe the importance of regular physical fitness examinations for firefighters because firefighters overestimated their physical fitness with their self-assessment compared to the objectively measured physical fitness level. On the other hand, physical training has a cardioprotective effect on firefighters^17^. Despite the background that the combination of cardiovascular risk factors and extreme physical workload can lead to cardiovascular complications, it is essential to assess physical performance objectively in this occupational group.

Therefore, the present study investigates whether there is a relationship between cardiorespiratory fitness and cardiovascular risk factors in the group of firefighters.

## Methods

### Study population

We enrolled ninety-seven male German firefighters in this cross-sectional study. The inclusion criteria for firefighters was full-time work at a fire department in active emergency service. The shift schedule characterized the working conditions. All participants are working in cities in the Westphalia area of Germany. Exclusion criteria were to be over 60, not working in shift schedule, and working in the administrative outside the active emergency service.

The study was carried out by Helsinki’s declaration rules and approved by the Witten/Herdecke Ethics Committee. We obtained written, informed consent from all participants.

We split study examinations over 2 days. On the first day, the anthropometric characteristics, history of the probands, resting blood pressure and resting heart rate measurement, and the blood sample, were taken. We performed spiroergometry exercise testing on the second day.

### Assessment of cardiorespiratory fitness

Spiroergometry testing involved exercising on a cycle ergometer (Ergo_bike premium 8i, Daum Electronics GmbH) with electrocardiogram monitoring and estimation of maximal oxygen consumption (V_O2max_) (Measurement system: Metalyzer 3B, Cortex Biophysik GmbH). We performed spiroergometry exercise testing in the following manner: Start at 50 watts and continuously increasing by 25 watts every 2 min (ramp test). The test ended when the subject could no longer maintain the predefined cadence of 80 revolutions per minute or if the proband was subjectively exhausted. There was no further increase in maximal oxygen uptake after 20 s. Another endpoint was an exceeding 85% of their maximum predicted heart rate defined as 220-age. During the exercise test, metabolic equivalents (METS) were recorded and analyzed for the present study. One MET was described to be 3.5 mL kg^−1^ min^−1^, often characterized as the metabolic cost of resting quietly^18^.

We categorized the participants for each working group into four groups based on the achieved METS. Representing very low (≤ 10 METS), low (10 to 12 METS), intermediate (> 12 to 14 METS), and high fitness levels (> 14 METS).

#### Assessment of cardiovascular risk factors

This study questionnaire collected individual use of tobacco (cig./day) and years of professional experience. Bodyweight, body fat (%) and body composition were determined using the Tanita BC-418MA Segmental Body Composition Analyzer (Tanita Corporation, Japan). We instructed participants to wear only comfortable shorts with no other clothing or shoes. We measured height in a standing position with a clinic stadiometer. Body mass index (BMI) calculation was defined as the weight in kilograms divided by the square of height in meters. The measurement of diastolic (RDBP) and systolic (RSBP) resting blood pressure was performed in a supine position with calibrated standard blood pressure cuffs. Before the first blood pressure and heart rate measurement, we served a 5-min break in a supine position. We measured blood pressure three times at intervals of 1 min. A mean value was formed from these three recorded blood pressure measurements, given the resting blood pressure value.

Resting a 12-channel-ECG measured heart rate. Waist circumference was determined at the end of expiration in a standing position in the center of the lower edge of the ribs and the iliac crest's upper edge.

Participants fasted (refrain from eating and drinking for 4 to 6 h) before having blood examinations in the morning. Fasting venous blood sample parameters included: total cholesterol, high-density lipoprotein (HDL), low-density lipoprotein (LDL), triglyceride.

### Statistical analysis

The statistical analysis occurred by Stata/IC 13.1 for Windows (StataCorp LP, College Station, TX). Baseline characteristics were categorized by using mean, standard deviations (SD), and medians. The frequency was reported for categorical variables. Analysis of variance was performed for quantitative variables, whereas the x^2^ test was used for group comparison for categorical variables. We described the influence of METS and the influence of METS and BMI on CVD risk factors using linear regression models. We performed different models (unadjusted, adjusted for age, adjusted for age, and BMI). All statistical tests were two-sided with a significance level of 0.05.

### Ethics approval and consent to participate

The study was approved by the human research ethics committee at University of Witten/Herdecke. All subjects provided written informed consent.

### Consent for publication

All the authors listed have approved the manuscript for publication.

## Results

### Baseline characteristics

The average age of the examined firefighters was 40.5 years (SD 9.0.) and the average BMI was 25.9 kg/m^2^ (SD 3.2). Based on the international BMI classification, 41.2% of the firefighters were overweight, and 10.3% were obese. 48.5% were of normal weight. The average waist circumference was 89.8 cm (SD 10.0) and was thus above the reference value of 84 cm of the International Diabetes Foundation. Cardiorespiratory fitness measured in METS averaged 10.7 (SD 1.7) in the study population. In most cases (40.2%), the firefighters achieved > 10 ≤ 12 METS, > 12 to ≤ 14 was achieved by 18.6% of the study population. There was a large variance in cigarette consumption (cigarettes per day) with an average of 2.6 (SD 6.3) (min–max: 0.0–20.0). Professional experience varies between 2.5 and 37 years with an average of 16.3 years (SD 9.1). The baseline characteristics of the study population are summarized in Table 1.

**Table 1:** Baseline characteristics of the participants.

Variables	n	Mean	SD	Median	Min–max
Age	97	40.5	9.0	41.0	23–58
BMI (kg/m^2^)	97	25.9	3.2	25.1	18.8–35.8
METS (absolut)	97	10.7	1.8	10.8	6.6–15.6
Tobacco use (Cigarettes/day)	97	2.6	6.3	0.0	0.0–20.0
Body fat (%)	97	17.7	6.2	17.5	2.9–35.9
Waist circumference (cm)	97	89.8	10.0	90.0	62–117
Professional experience (years)	97	16.3	9.1	15.0	2.5–37.0

*BMI* Body mass index, *METS* metabolic equivalents.

The associations of cardiovascular risk factors and cardiorespiratory fitness category are summarized in Table 2. These results are unadjusted and only adjusted for age as well as adjusted for BMI. Overall, higher lipid values, higher RDBP, and RSBP values, higher heart rates, higher waist circumference, higher body fat percentages, and increasing years of work experience are inversely related with inversely to lower cardiorespiratory fitness levels. The unadjusted analysis describes significant associations for higher cardiorespiratory fitness with lower BMI (p ≤ 0.0001), lower waist circumference (p ≤ 0.0001), body fat percentage (p ≤ 0.0001), RSBP (p = 0.0061), lower triglycerides (p = 0.0018) and total cholesterol levels (p = 0.0443). After adjusting for age, there are significant differences between higher cardiorespiratory fitness and lower BMI (p ≤ 0.0001) and abdominal waist circumference (p ≤ 0.0001), lower body fat percentage (p ≤ 0.0001), lower RSBP (p ≤ 0. 0092) and lower triglyceride values (p ≤ 0. 0078). Adjusted for age and BMI there was a significant association between higher cardiorespiratory fitness and lower body fat percentage (p ≤ 0. 0040).

**Table 2:** Cardiovascular risk factors of each METS category.

	METS ≤ 10 n = 38	10 < METS ≤ 12 n = 39	12 < METS ≤ 14 n = 18	METS > 14 n = 2	p1	p2	p3
Age	44.0 (8.3)	39.3 (9.0)	37.2 (8.4)	29.0 (2.8)	0.0082	n/a	n/a
BMI, kg/m^2^	27.7 (3.6)	25.2 (2.1)	23.7 (2.3)	22.8 (0.7)	< 0.0001	< 0.0001	n/a
Waist circumference, cm	95.0 (11.1)	89.2 (6.3)	81.6 (7.8)	77.2 (0.4)	< 0.0001	< 0.0001	0.1429
Body fat, %	21.8 (5.7)	16.5 (4.0)	12.5 (5.3)	8.9 (7.1)	< 0.0001	< 0.0001	0.0040
RSBP, mmHg	127.9 (10.9)	127.6 (9.6)	121.9 (5.7)	115.0 (7.1)	0.0061	0.0092	0.0650
RDBP, mmHg	86.5 (8.2)	83.2 (6.7)	82.2 (5.5)	75.0 (7.1)	0.0564	0.1902	0.5897
HR, bpm	71.0 (12.3)	66.3 (10.2)	64.6 (13.5)	53.5 (6.4)	0.1169	0.1173	0.0932
Triglycerides, mg/dL	161.1 (78.1)	144.2 (79.0)	104.9 (42.6)	77.5 (29.0)	0.0018	0.0078	0.0994
Total cholesterol, mg/dL	206.6 (35.7)	198.7 (34.6)	185.1 (26.7)	189.5 (41.7)	0.0443	0.3322	0.4832
HDL-cholesterol, mg/dL	52.3 (11.4)	55.2 (11.8)	60.1 (11.6)	79.0 (33.9)	0.0648	0.0661	0.3482
LDL-cholesterol, mg/dL	122.1 (30.3)	115.6 (33.7)	104.0 (28.5)	95.0 (1.4)	0.0982	0.4648	0.6136
Professional experience, years	19.6 (9.0)	14.8 (9.1)	13.7 (7.7)	6.5 (0.7)	0.0209	0.7276	0.7239

p1: unadjusted; p2: adjusted for age; p3: adjusted for age and BMI.

*BMI* body mass index, *HR* heart rate, *RDBP* diastolic resting blood pressure, *RSBP* systolic resting blood pressure.

The study participants were divided into four groups, taking into account cardiorespiratory fitness and BMI: “low fit (METS ≤ 12) and obese (BMI ≥ 30), fit (METS > 12) and obese (BMI ≥ 30), low fit (METS ≤ 12) and not obese (BMI < 30) and fit (METS > 12) and not obese (BMI < 30). Only one participant could be assigned to the fit and obese group. Therefore, no valid statement can be assigned to this group, and for better clarity, this group is not shown in the corresponding table.

Most study participants were “low fit and not obese” (n = 47) followed by “low fit and obese” (n = 30). A comparison of the four groups shows significant differences for all examined cardiovascular risk factors (all parameter p < 0.05) (Table 3). After adjustment for age, significant differences remain in the comparison for the four groups for body fat percentage (p ≤ 0.0001), RSBP (p = 0.0014), and triglycerides (p = 0.0006) and HDL-cholesterol values (p = 0.0021).

**Table 3:** Groups divided by METS and BMI (Mean and SD).

	Low fit and obese n = 30	Low fit and not obese n = 47	Fit and not Obese n = 19	p1	p2
Age	45.5 (7.0)	39.1 (9.2)	35.5 (7.8)	< 0.0001	n/a
BMI, kg/m^2^	29.1 (3.1)	24.8 (1.9)	23.5 (2.1)	–	–
Waist circumference, cm	101.0 (6.2)	86.4 (6.0)	80.3 (6.6)	–	–
Body fat, %	23.6 (4.4)	16.2 (4.2)	12.0 (5.5)	< 0.0001	< 0.001
RSBP, mmHg	128.9 (11.5)	127.1 (9.3)	120.8 (5.8)	0.0007	0.0014
RDBP, mmHg	86.6 (8.5)	83.7 (6.9)	81.6 (6.0)	0.0592	0.2998
HR, bpm	69.9 (12.2)	67.8 (11.0)	63.4 (13.7)	0.2396	0.2708
Triglycerides, mg/dL	190.8 (84.8)	128.1 (63.9)	104.1 (41.9)	< 0.0001	0.0006
Total cholesterol, mg/dL	213.6 (33.5)	195.6 (34.7)	185.7 (27.8)	0.0066	0.2641
HDL-cholesterol, mg/dL	49.8 (9.9)	56.4 (12.0)	61.2 (14.6)	0.0036	0.0021
LDL-cholesterol, mg/dL	127.4 (30.9)	113.6 (31.9)	103.6 (27.8)	0.0209	0.4713
Professional experience, year	20.9 (8.4)	14.8 (9.1)	12.2 (7.0)	0.0004	0.7635

p1: unadjusted; p2: adjusted for age.

*BMI* body mass index, *HR* heart rate, *RDBP* diastolic resting blood pressure, *RSBP* systolic resting blood pressure.

We investigated the relationship between cardiovascular risk factors and cardiorespiratory fitness (METS) as continuous variables using the linear regression model. We present the results in Table 4. In the unadjusted model, every increase in METS is associated with a significant decrease in all examined cardiovascular risk factors. After adjustment for age, there was still a significant difference in all examined cardiovascular risk factors except for total cholesterol, LDL-cholesterol, and work experience. When adjusted for age and BMI, the values were only significantly lower for the body fat percentage (p = 0.0061). The effects of fitness levels on the waist circumference and body fat% were highly significant in the unadjusted and adjusted analysis for age (p < 0.0001). In contrast, the effect was harmonized after additional adjustment for BMI.

**Table 4:** Linear regression models of cardiovascular risk factors and METS as continuous variable.

Variables	Model 1		Model 2		Model 3	
	Beta (SE)	p	Beta (SE)	p	Beta (SE)	p
Age	− 2.04 (0.43)	< 0.0001	n/a	n/a	n/a	n/a
BMI, kg/m^2^	− 1.07 (0.17)	< 0.0001	− 1.02 (0.17)	< 0.0001	n/a	n/a
Waist circumference, cm	− 3.23 (0.55)	< 0.0001	− 2.62 (0.60)	< 0.0001	− 0.66 (0.67)	0.3289
Body fat, %	− 2.20 (0.31)	< 0.0001	− 1.93 (0.34)	< 0.0001	− 0.81 (0.29)	0.0061
RSBP, mmHg	− 1.58 (0.58)	0.0072	− 1.59 (0.59)	0.0085	− 1.23 (0.67)	0.0719
RDBP, mmHg	− 1.36 (0.41)	0.0014	− 1.10 (0.45)	0.0159	− 0.83 (0.53)	0.1262
HR, bpm	− 2.01 (0.86)	0.0219	− 2.08 (0.86)	0.0178	− 2.52 (0.88)	0.0051
Triglycerides, mg/dL	− 12.38 (3.97)	0.0024	− 11.61 (4.46)	0.0108	− 7.27 (5.25)	0.1692
Total cholesterol, mg/dL	− 4.90 (1.77)	0.0067	− 2.16 (1.95)	0.2700	− 1.75 (2.37)	0.4631
HDL-cholesterol, mg/dL	2.03 (0.93)	0.0320	2.11 (0.98)	0.0351	1.52 (1.17)	0.1956
LDL-cholesterol, mg/dL	− 4.41 (1.50)	0.0042	− 1.98 (1.64)	0.2296	− 1.70 (1.97)	0.3921
Professional experience, years	− 1.77 (0.49)	0.0004	0.11 (0.26)	0.6686	0.18 (0.26)	0.4793

Model 1: linear regression, unadjusted.

Model 2: linear regression, adjusted for age.

Model 3: linear regression, adjusted for age and BMI.

*BMI* body mass index, *HR* heart rate, *RDBP* diastolic resting blood pressure, *RSBP* systolic resting blood pressure.

## Discussion

The present study of German firefighters shows a strong association between low cardiovascular risk parameter profiles and good cardiorespiratory fitness. Specifically, we demonstrated that after adjustment for age, there were significantly lower values for BMI, waist circumference, body fat percentage, RSBP and triglycerides with increased cardiorespiratory fitness. Overall, there is broad evidence that physical activity positively influence cardiovascular risk factors^19,20^. Evidence of a positive effect of cardiorespiratory fitness on cardiovascular risk factors is of key importance to avoid cardiovascular complications. Only Carey et al.^21^, described eight per cent of the examined firefighters complained of a lack of physical well-being. Several studies have already proven the importance of physical activity for firefighters. Studies to date have shown that low physical activity in firefighters is associated with a high cardiovascular and metabolic risk profile^22–24^. Our study was able to prove this assumption for German firefighters as well. Physically efficient firefighters have increased physical fitness and energy, which allows them to do their job more efficiently and safely compared to those who do not possess high physical capacity^25^. However, in our study only 21.7% (n = 20) had METS number of > 12 and 39% (N = 38) of all study participants had METS number of ≤ 10. The low number of high METS achieved is probably the high rate owed to overweight or obese study participants. After all, more than half of the study participants had a BMI > 25 and only one study participant is assigned to the “Fit and Obese” group. Increased BMI values are associated with decreased cardiorespiratory fitness, this fact could also be found in another study by Nogueira et al.^26^. In this study, Brazilian firefighters showed an increased BMI that also correlates strongly with a decreased cardiorespiratory fitness. Overweight firefighters in poor health are also more at risk of gaining weight than those of normal weight^27^. Moreover, American firefighters’ study results showed significantly better cardiorespiratory fitness and fewer cardiovascular risk factors in normal-weight firefighters compared to overweight firefighters^28^. The enormous importance of cardiorespiratory fitness with fat metabolism parameters in firefighters is confirmed not least by a study by Baur et al.^24^. In this study, metabolic syndrome was inversely related to cardiorespiratory fitness levels. Nevertheless, other occupational groups, e.g. office workers, are at higher risk for cardiovascular and metabolic disorders^29,30^.

These results should be viewed critically considering the background that firefighters are required to demonstrate solid physical fitness through sporting activity, even during working hours. Anomalies during examinations were often found in subjectively healthy firefighters with low physical fitness^31^.

However, firefighters’ physical fitness seems elementary, as they often reach their physical performance limits during emergency operations. Their heavy protective equipment requires an additional physical strain. Stressors to exposed firefighters are strenuous physical work, transport and heavy equipment, dangerous surroundings, extreme temperatures, toxic gases and substances, and intense psychological stress in emergencies. All of these factors require good physical performance, endurance, and resistance. Drew-Nord et al.^3^ investigated American firefighters for cardiorespiratory fitness in their study and described a relative oxygen uptake (rel. V_O2max_) of 33.6 mL/kg^−1^ min^−1^ to 46 mL/kg^−1^ min^−1^ as necessary. In our previous studies, we reported the relative V_O2max_ of German firefighters; the values amounted to a range of 37.3 mL/kg^−1^ min^−1^^32,33^. Studies by Durand et al.^23^ pointed out that only about 20% of examined professional firefighters performed aerobic endurance training of > 150 min a week. We can resume that the subjects in our study with higher cardiorespiratory fitness, characterized by the “Fit and not Obese” study group, had significantly (after adjustment for age) lower body fat percentages, RSBP, and triglyceride values.

In our study group, we were also able to show that increased cardiorespiratory fitness also influenced the reduction in RDBP. It is fascinating because epidemiological studies showed that even small blood pressure changes could reduce the rate of cardiovascular diseases and strokes^34,35^.

Continuous health-promoting programs are necessary to increase cardiovascular fitness^36^. Firefighters participating in health promotion programs have a higher maximum oxygen intake than their counterparts without these programs. Regular physical training in firefighters increases efficiently aerobic capacity—by up to 20%^37^.

## Limitations

In this study, we measured cardiorespiratory fitness using a definition of METS in connection to exercise spiroergometry. These methods are recommended by Wassermann et al. and are well accepted in sports medicine^18^. Another powerful and adequate method we couldn't consider in this study is to measure V_O2peak_ using a metabolic measurement system to assess cardiorespiratory fitness^38^.

## Conclusions

Our study's results clarify that increasing cardiorespiratory fitness is a fundamental starting point for the reduction of cardiovascular risk factors and the prevention of cardiovascular complications in firefighters. Our study demonstrated central risk factors as BMI, waist circumference, RSBP, and triglyceride values. Accordingly, it is essential to establish training plans to prevent and reduce cardiovascular risk factors, which specifically aims to increase cardiorespiratory fitness. Previous study results indicate that health-promoting programs have already proven themselves in firefighters and that cardiorespiratory fitness testing is a useful parameter for assessing the cardiovascular risk in firefighters. Therefore, it is essential to establish extensive prospective studies to understand better cardiorespiratory fitness protective effect on cardiovascular risk factors in firefighters or other emergency services to protect these important occupational groups of public safety from cardiovascular events.

## Data Availability

The datasets analysed during the current study are available from the corresponding author on reasonable request.

## Abbreviations

BMI: Body mass index
HR: Heart rate
METS: Metabolic equivalents
Rel. VO2max: Relative maximal oxygen uptake
RDBP: Diastolic resting blood pressure
RSBP: Systolic resting blood pressure
